# Eye-tracking studies on developmental language disorder: a systematic review

**DOI:** 10.3389/fpsyg.2026.1862020

**Published:** 2026-09-09

**Authors:** Ziteng Liu, Qihang Jiang

**Affiliations:** 1 School of English, University of Nottingham, Nottingham, United Kingdom; 2 School of Humanities & Languages, University of New South Wales, Sydney, NSW, Australia

**Keywords:** cognitive processing, developmental language disorder (DLD), eye-tracking, language development, language processing, linguistic domains, systematic review

## Abstract

Developmental language disorder (DLD) is a neurodevelopmental condition characterized by persistent difficulties in language acquisition and processing. While traditional behavioral and neurophysiological methods have provided valuable insights, eye-tracking has emerged as a powerful tool for capturing real-time cognitive processing during language comprehension. However, evidence from eye-tracking studies in DLD remains underrepresented and fragmented across linguistic domains and experimental paradigms. This systematic review synthesizes 33 empirical studies and examines how eye-tracking has been used to investigate real-time language processing in children with DLD. The review focuses on three key dimensions: (a) cognitive-linguistic domains addressed, (b) paradigms and eye-movement metrics employed, and (c) recurring processing differences between children with DLD and typically developing (TD) counterparts. The synthesis reveals three broadly consistent processing patterns in DLD: slower processing efficiency, reduced predictive processing, and increased reliance on semantic cues over morphosyntactic information. These patterns suggest that language difficulties in DLD are not solely attributable to representational deficits but are closely linked to inefficiencies in real-time integration and competition resolution during language processing. By consolidating findings across paradigms, this review provides a cognitively grounded account of DLD and highlights the role of eye-tracking in uncovering underlying processing mechanisms. Implications for theoretical models of language processing and future methodological directions are discussed.

## 1 Introduction

Developmental language disorder (DLD) is a neurodevelopmental condition in which individuals, typically from early childhood, exhibit persistent deficits in the acquisition, understanding, production, or use of language (World Health Organization, 2025). This condition is distinguished from language disorders caused by other biomedical conditions (e.g., Autism Spectrum Disorder) (Bishop et al., 2017). Individuals with DLD may experience impairment in terms of syntax, morphology, word finding, phonology, pragmatics, discourse, and verbal learning/memory domains (Bishop et al., 2017).

Due to deficits in language and communication, DLD may have a persistent impact on individuals’ educational, social, and emotional development (Ekström et al., 2023); hence, this condition can detrimentally affect affected individuals’ social behaviors and educational progress (Law et al., 2009). These difficulties are likely to persist into adulthood, affecting individuals’ postschool employment rate (Law et al., 2009; Conti-Ramsden and Durkin, 2012). Previous researchers have estimated the prevalence of DLD to be 7.58% (Norbury et al., 2016). In the United Kingdom, for instance, two children in every average classroom of 30 have DLD (Speech and Language UK, n.d.). However, this condition receives less attention than other neurodevelopmental disorders in scholarly communities. According to McGregor (2020), although 5,461,200 confirmed cases of DLD were reported in the United States in 2019, only 1,388 publications on DLD were identified between 2010 and 2019. By contrast, despite the relatively smaller number of confirmed ASD cases during the same year (479,700), the number of ASD-related publications from the same period reached 38,110. Furthermore, publications on DLD in the UK were also relatively scarce during that time (McGregor, 2020). These patterns suggest that DLD is a relatively prevalent yet underestimated disorder that can have lasting impacts on individuals’ lives.

Experimental studies have used a range of methodologies, each offering distinctive advantages and limitations for exploring the language processing and production mechanisms of children with DLD. Researchers often employ a task-based approach to directly observe linguistic features of children with DLD. For instance, Leonard et al. (1997) employed specific play activities and constructed probes to examine the use of English grammatical morphemes in children with and without DLD. Although this approach is non-invasive, the examination of real-time linguistic processing through this method is not feasible. Instead, event-related brain potentials (ERPs) can address this issue. This technology allows researchers to observe online neurocognitive processes involved in linguistic processing in a non-invasive manner (Royle and Courteau, 2014). For instance, Kornilov et al. (2015) employed a cross-modal picture-word matching paradigm combined with ERPs to investigate lexical and phonological processing in children with DLD. While ERPs can provide real-time processing data in linguistic experiments, such technology can be intimidating for young participants and is sensitive to movement artifacts. Moreover, ERPs primarily display temporal information and do not capture how children allocate visual attention to linguistic input in naturalistic contexts. To address these limitations, researchers have increasingly turned to eye-tracking technology, a child-friendly tool that records gaze patterns during listening or reading tasks. This technology provides an experimental environment that closely resembles natural conditions. Furthermore, quantified eye-movement indices can be collected in real time, reflecting both conscious and unconscious processing (Conklin and Pellicer-Sánchez, 2016; Huettig et al., 2011). For example, Christou et al. (2022a) utilized an eye-tracker to examine verbal tense morphology comprehension in Spanish- and Catalan-speaking children with DLD. The same research team (Coloma et al., 2024) has also used this technology to examine article comprehension ability in a group of monolingual Spanish-speaking children with and without DLD at two different time points.

Despite rapid growth in eye-tracking research on language development, evidence specifically targeting children with DLD is scattered across paradigms, linguistic domains, and analytic choices, hampering cumulative inference. No systematic review has consolidated how eye-tracking has been used to examine real-time language processing in DLD. The present review systematically maps this literature by: (a) summarizing findings by linguistic domain, (b) cataloging paradigms and metrics, and (c) evaluating consistencies in DLD-TD differences and methodological gaps to guide future work.

This synthesis offers an integrated understanding of the potential of eye-tracking as a tool for studying children with DLD. Furthermore, the synthesis aims to inform both theoretical models of language processing and applied directions for assessment and intervention. The predefined questions of this systematic review were as follows: (i) the linguistic domains of language processing in DLD examined using eye-tracking and the main findings; (ii) the paradigms and eye-movement metrics employed and the relationship between these methodological choices and the reported effects; and (iii) the extent to which eye-tracking studies reveal consistent differences between DLD and typically developing peers (TDs) in real-time processing.

## 2 Methodology

The present systematic review was conducted in accordance with the Preferred Reporting Items for Systematic Reviews and Meta-Analyses (PRISMA) 2020 statement. The review protocol defined the research strategy, eligibility criteria, screening procedures, and data extraction framework in advance. The review was not preregistered in PROSPERO due to its primary focus on cognitive-linguistic mechanisms and eye-tracking evidence in developmental language disorder rather than intervention effectiveness, diagnostic accuracy, or clinical treatment outcomes.

### 2.1 Search strategy

To ensure the comprehensiveness of the present systematic review, five databases were used to collect eye-tracking studies on children with DLD. These databases cover a wide range of peer-reviewed journal articles. In addition, these databases allow researchers to find articles by time range. These features enabled the present review to include peer-reviewed, empirical eye-tracking studies on children with DLD worldwide.

Databases searched:

(1). Scopus
(2). Web of Science
(3). PubMed
(4). ScienceDirect
(5). Google Scholar

Searches were conducted in August 2025 using Boolean combinations of keywords related to the population, method, and disorder terminology:

(“eye-tracking” OR “eye tracking”) AND (“language disorder*” OR “language impairment*” OR “developmental language disorder” OR “DLD” OR “specific language impairment” OR “SLI”) AND (“child*” OR “developmental”)

Before the term “developmental language disorder” gained institutional recognition, researchers lacked agreement about the terminology for children’s language problems (Bishop et al., 2017). To avoid neglecting relevant articles, “language disorder*” and “language impairment*” were used alongside “DLD” and “SLI.” Due to the breadth of the first two terms, “AND (“child*” OR “developmental”)” was applied to ensure the range of research subjects.

All retrieved records were exported into Zotero for reference management and deduplication.

### 2.2 Eligibility criteria

Eligibility was based on the PICO framework (see Table 1):

**TABLE 1:** PICO framework used in the present systematic review.

Component	Specifications
Population	Children and adolescents (0–18) diagnosed with DLD/SLI/suspected DLD
Intervention	Eye-tracking used as a primary data collection tool
Comparison	Typically developing (TD) controls or other comparison groups
Outcome	Language processing or production
Time frame	2000–2025
Language	English

Inclusion criteria:

- Journal articles utilizing eye-tracking technology to examine language processing and production mechanisms of participants with DLD.
- Empirical, peer-reviewed journal articles.
- Articles written in English.
- Studies using alphabetic languages (e.g., English, Spanish, Catalan).

Exclusion criteria:

- Journal articles focusing exclusively on linguistic features of children with DLD without using eye-tracking technology as data-collection method.
- Studies focused on non-linguistic features of children with DLD.
- Studies focused on irrelevant disorders and/or irrelevant groups.
- Non-eye-tracking research.
- Non-peer-reviewed, non-empirical, and non-original studies.

Screening process:

The initial dataset consisted of 635 peer-reviewed journal articles and was uploaded to Zotero for deduplication. After further screening, the remaining 36 full-text articles were assessed for eligibility. The present review had a relatively high exclusion rate due to the intentionally broad search strategy adopted to maximize sensitivity and capture studies using both contemporary and historical terminology (e.g., SLI and DLD). Therefore, a substantial amount of records were identified during the initial stage.

A total of 445 studies were excluded based on inclusion/exclusion criteria.

A total of 36 studies were retrieved for full-text screening.

After full-text assessment, 33 studies met all inclusion criteria.

**FIGURE 1:**
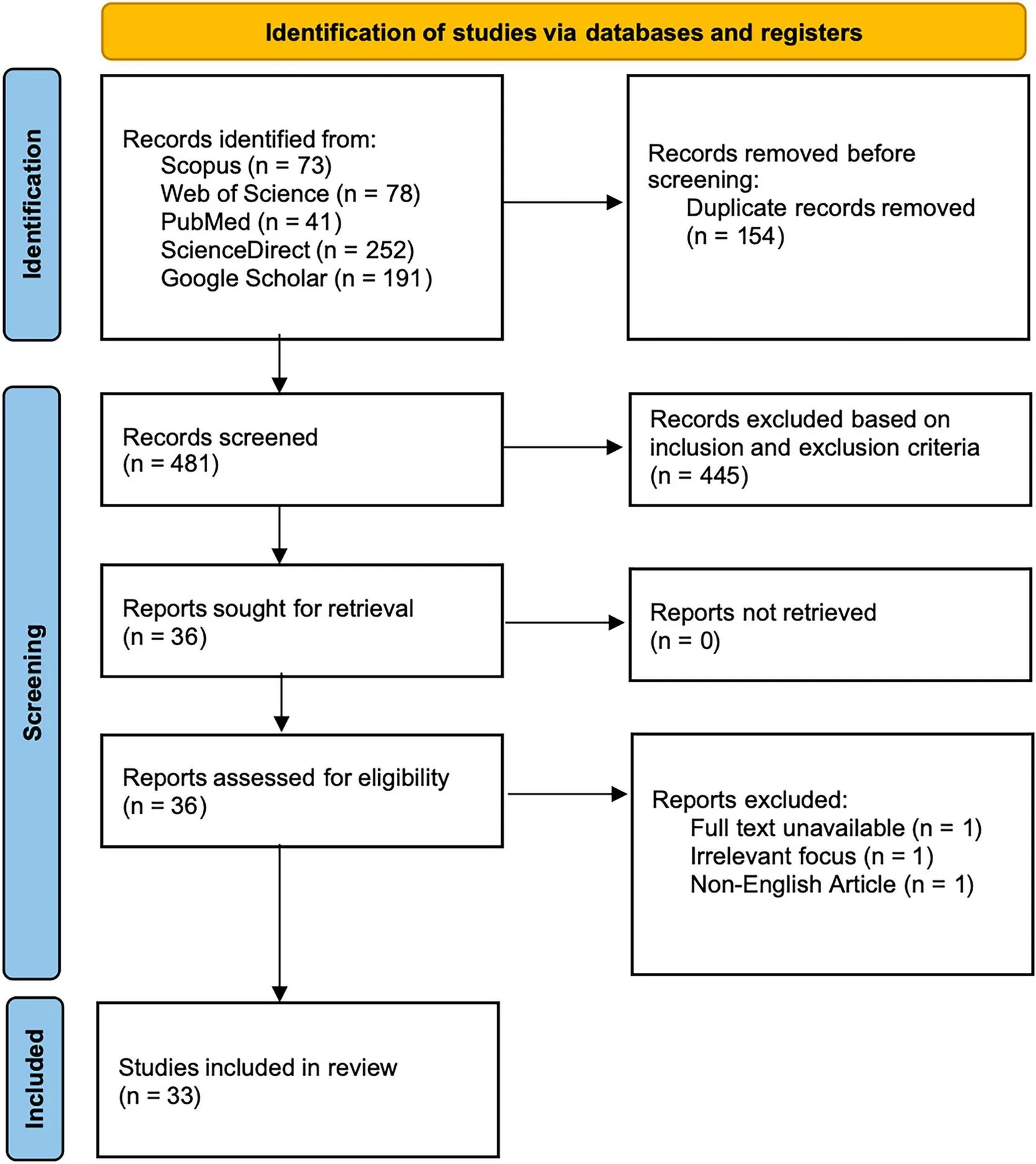
Flowchart of the selection of published eye-tracking studies on developmental language disorder.

Screening was independently completed by two reviewers (Ziteng and Qihang). Any disagreements between the two reviewers regarding study eligibility were resolved through discussion until consensus was reached, in accordance with the predefined inclusion and exclusion criteria and after careful review of the article’s abstract, methodology, discussion, and conclusion sections. Specifically, an additional three records, distinct from the three reports excluded during the full-text eligibility assessment, were excluded through this process before full-text screening. Figure 1 presents the PRISMA 2020 flow diagram displaying the process of article selection.

### 2.3 Data extraction

The structured data extraction sheet was based on Microsoft Excel and was developed with reference to Toki (2024) and Hu and Aryadoust (2024).

A total of 33 eligible journal articles’ data were collected using the data extraction sheet. The sheet covered articles’ fundamental information such as year of publication, title of the journal, and DOI/URL. In addition, the sheet also extracted articles’ publication range, country/region distribution, DLD diagnosis, and exclusion criteria. The sheet also contained high-level descriptions of populations (e.g., TD vs. DLD). The sheet also extracted data on eye-tracking devices, defined Areas of Interest (AOIs), and reported metrics. Unlike typical eye-tracking studies on linguistics, these studies focusing on DLD tended to adopt the Visual World Paradigm (VWP). Such a paradigm primarily requires participants to observe pictures of objects while listening to corresponding audio. In this sense, rather than recording metrics such as fixation duration, these studies mainly recorded participants’ proportion of looks.

The sheet possessed a quality appraisal section for eligible studies. The appraisal process was conducted by two reviewers (Ziteng and Qihang). These reviewers evaluated an article based on sampling adequacy, addressed confounders, etc. Methodological quality was assessed using an adapted version of the Mixed Methods Appraisal Tool (MMAT; Hong et al., 2018). Overall, most included studies met several MMAT criteria; however, recurring methodological limitations were identified in sampling representativeness, reporting transparency, and the control of confounding variables. The item-level MMAT appraisal results are provided in the data extraction sheet in the Supplementary material.

A formal meta-analysis was not conducted due to substantial heterogeneity across studies regarding linguistic domains, participant characteristics, eye-tracking paradigms, analytical approaches, and statistical reporting practices. Many studies incorporated distinct eye-movement metrics and analysis windows, which limited the comparability of effect sizes across experiments. Therefore, a narrative synthesis approach was considered more appropriate than a quantitative aggregation for the current review.

## 3 Findings

### 3.1 Overview of included evidence

The present systematic review included 33 empirical studies. The fundamental information of these articles is displayed in Table 2.

**TABLE 2:** Studies included in the present systematic review.

No.	References	Title	Journal	Year of publication
1	McMurray et al., 2010	Individual differences in online spoken word recognition: Implications for SLI	Cognitive Psychology	2010
2	Andreu et al., 2011	Narrative comprehension and production in children with SLI: An eye movement study	Clinical Linguistics and Phonetics	2011
3	Palmović and Jušić, 2011	Anticipatory gaze and argument structure building in children with specific language impairment	Acta Linguistica Hungarica	2011
4	Andreu et al., 2012	Auditory word recognition of nouns and verbs in children with Specific Language Impairment (SLI)	Journal of Communication Disorders	2012
5	Andreu et al., 2013a	The formulation of argument structure in SLI: an eye-movement study	Clinical Linguistics and Phonetics	2013
6	Pons et al., 2013	Perception of audio-visual speech synchrony in Spanish-speaking children with and without specific language impairment	Journal of Child Language	2013
7	Borovsky et al., 2013	Lexical activation during sentence comprehension in adolescents with history of specific language impairment	Journal of Communication Disorders	2013
8	Andreu et al., 2013b	Anticipatory sentence processing in children with specific language impairment: Evidence from eye movements during listening	Applied Psycholinguistics	2013
9	Ellis et al., 2015	Novel word learning: An eye-tracking study. Are 18-month-old late talkers really different from their typical peers?	Journal of Communication Disorders	2015
10	Mak et al., 2017.	Connective processing by bilingual children and monolinguals with specific language impairment: distinct profiles	Journal of Child Language	2017
11	McMurray et al., 2019	A real-time mechanism underlying lexical deficits in developmental language disorder: Between-word inhibition	Cognition	2019
12	Christou et al., 2020	Real time comprehension of Spanish articles in children with developmental language disorder: Empirical evidence from eye movements	Journal of Communication Disorders	2020
13	Chung and Yim, 2020	Quick incidental learning of words by children with and without specific language impairment: An eye-tracking study	Communication Sciences & Disorders	2020
14	Moreno-Pérez et al., 2020	Comprehending reflexive and clitic constructions in children with autism spectrum disorder and developmental language disorder	International Journal of Language and Communication Disorders	2020
15	van Alphen et al., 2021	Word Recognition and Word Prediction in Preschoolers With (a Suspicion of) a Developmental Language Disorder: Evidence From Eye Tracking	Journal of Speech, Language, and Hearing Research	2021
16	Christou et al., 2021	Real-time comprehension of Spanish prepositions and prepositional locutions in bilingual children with developmental language disorder: A study based on eye-movement evidence	International Journal of Language and Communication Disorders	2021
17	Ahufinger et al., 2021	Cross-situational statistical learning in children with developmental language disorder	Language, Cognition and Neuroscience	2021
18	Christou et al., 2022b	Online comprehension of verbal number morphology in children with developmental language disorder: An eye-tracking study	Journal of Speech, Language, and Hearing Research	2022
19	Helo et al., 2021	Objects shape activation during spoken word recognition in preschoolers with typical and atypical language development: An eye-tracking study	Language Learning and Development	2021
20	Christou et al., 2022a	Insights from real-time comprehension of Spanish verbal tense in children with developmental language disorder: An eye-tracking study	Applied Psycholinguistics	2022
21	Curtis et al., 2023b	Specificity of phonological representations in U.S. English-speaking late talkers and typical talkers	Infancy	2023
22	Curtis et al., 2023a	Sensitivity to Semantic Relationships in U.S. Monolingual English-Speaking Typical Talkers and Late Talker	Journal of Speech, Language, and Hearing Research	2023
23	Broedelet et al., 2023	Implicit cross-situational word learning in children with and without developmental language disorder and its relation to lexical-semantic knowledge	Frontiers in Communication	2023
24	LaTourrette et al., 2023	From Recognizing Known Words to Learning New Ones: Comparing Online Speech Processing in Typically Developing and Late-Talking 2-Year-Olds	Journal of Speech, Language, and Hearing Research	2023
25	Horvath and Arunachalam, 2023	Assessing receptive verb knowledge in late talkers and autistic children: advances and cautionary tales	Journal of Neurodevelopmental Disorders	2023
26	Lara-Díaz et al., 2024	Visual attention and phonological processing in children with developmental language disorder	Frontiers in Communication	2024
27	Kueser et al., 2024	Verb Vocabulary Supports Event Probability Use in Developmental Language Disorder	Journal of Speech, Language, and Hearing Research	2024
28	Guerra et al., 2024	Lexical-semantic processing in preschoolers with Developmental Language Disorder: an eye tracking study	Frontiers in Psychology	2024
29	Coloma et al., 2024	Article comprehension in monolingual Spanish-speaking children with developmental language disorder: A longitudinal eye tracking study	International Journal of Speech-Language Pathology	2024
30	Giberga et al., 2025b	Prosody and gestures help pragmatic processing in children with Developmental Language Disorder	Journal of Communication Disorders	2025
31	Bahn et al., 2025	Processing of scene-grammar inconsistencies in children with developmental language disorder— insights from implicit and explicit measures	Brain Sciences	2025
32	Cholewa et al., 2025	Predictive use of grammatical gender during noun phrase decoding: An eye tracking study with German children with developmental language disorder	Journal of Speech, Language, and Hearing Research	2025
33	Giberga et al., 2025a	How children with and without developmental language disorder use prosody and gestures to process phrasal ambiguities	languages	2025

As shown in Table 2, the frequency of related articles published after 2020 has increased significantly compared to previous periods. In addition, these articles demonstrate a wide geographic distribution. The majority of these articles are based in European countries or regions, followed by those in the Americas and Asia. Eight alphabetic languages are examined in these studies. The geographic distribution of the included articles is presented in Table 3.

**TABLE 3:** Geographic distribution of the present systematic review.

Country/region	Language	*N*
Spain and Catalonia	Spanish, Catalan, or both	13
United States	English	9
Chile	Spanish	3
Germany	German	2
Netherlands	Dutch	2
Colombia	Spanish	1
Croatia	Croatian	1
Russia and Netherlands	Russian	1
The Republic of Korea	Korean	1

Mak et al. (2017) conducted the experiments in both Russia and Netherlands.

Participant characteristics for each study are summarized in Table 4, including sample size, age range, mean age of participants with DLD, and the proportion of female participants. The table also reports the trackloss rate for each study.

**TABLE 4:** Demographic information and trackloss rate in the present systematic review.

No.	Sample size (DLD)	Sample size (TD)	Other groups (*N*, label)	Age range	Mean age (DLD)	Sex (DLD, % female)	Trackloss rate (DLD, %)
1	20	40	(Non-specific-language-impaired: 17); (specific cognition impaired: 16)	ns	17 years, 2.9 months	ns	ns
2	12	12	–	ns	5 years, 5 months	50	ns
3	7	7	–	8–10 years	ns	43	ns
4	25	25 (age-matched)	(25, MLUw controlled group)	DLDs: 5 years, 3 months–8 years, 2 months; age controls: 5 years, 3 months–8 years, 2 months; MLUw controls: 3 years, 3 months–7 years, 1 month	6.69 years	28	6.59
5	11	11 (age-matched)	–	DLDs: 3 years, 8 months; age controls: 6 years, 6 months	5.28 years	55	8.32
6	20	20	–	TDs: 4 years, 4 months–6 years, 10 months; DLDs: 4 years, 4 months–7 years, 2 months	6.69 years	ns	ns
7	12	14	–	13.08–20.08 years	16.5 years	42	ns
8	25	25 (age-matched)	(25, MLUw controlled group); (31, adult group)	DLDs and age controls: 5 years, 3 months–8 years, 2 months; MLUw controlled group: 3 years, 3 months–7 years, 1 month	6.69 years	28	10.32
9	14	14	–	ns	18 months; 9.78 days	50	ns
10	20	29	(23, Russian-Dutch Bilinguals)	DLDs and TDs: 5 years, 2 months–6 years, 7 months; Bilinguals: 5 years, 0 months–6 years, 11 months	6 years, 3 months	40	ns
11	22	57	–	DLDs: 14 years, 7 months–18 years, 7 months; TDs: 14 years, 5 months–19 years, 11 months	16 years, 4 months	68	ns
12	24	24	(24, MLUw controlled group); (24, adult university students as a language-expert control group)	DLDs and TDs: 4 years, 6 months–12 years; MLUw controlled group: 4 years, 3 months–9 years, 4 months	7 years, 8 months	ns	ns
13	10	20	–	DLDs: 4 years, 0 months–5 years, 11 months; TDs: 4 years, 5 months–6 years, 2 months	5.11 years	ns	ns
14	15	15 (age-matched)	(15, language proficiency-matched); (15, ASD with high language proficiency, ASD HL); (13, ASD with low language proficiency, ASDLL)	DLDs: 7.9–14.70 years; Age controls: 7.7–18.06 years; language proficiency-matched: 3.10–13.00 years; ASD HLs: 7.20–18.10; ASD LLs: 6.61–17.61 years	10.44 years	40	ns
15	51	31	–	2.6–4.5 years	3.42 years	19.6	ns
16	24	24	(24, MLUw controlled group); (24, adult university students as a language-expert control group)	DLDs: 4.6–12.6 years; TDs: 4.6–12.2 years; MLUw controls: 4.6–9.4 years	7.8 years	ns	ns
17	38	38 (age-matched and sex-matched)	–	DLDs: 5.6–12.11 years; TDs: 5.7–12.9 years	8.7 years	32	ns
18	24	24	(24, MLUw controlled group); (24, adult group)	DLDs: 4 years, 6 months–12 years, 6 months; age controls: 4 years, 6 months–12 years, 2 months; MLUw controls: 4 years, 6 months–9 years, 4 months	7 years, 8 months	29	ns
19	20	22	(30, adult group)	DLDs and TDs: 5 years, 4 months–6 years, 6 months	5;9 years	30	ns
20	24	24	(24, MLUw controlled group); (24, adult group)	DLDs: 4.6–12.6 years; TDs: 4.6–12.2 years; MLUw controls: 4.6–9.4 years	7.8 years	ns	ns
21	22	23	–	Overall: 22–30 months	24.57 months	41	ns
22	21	24	–	Overall: 22–30 months	24.29 months	46	7.5 (overall)
23	26	26	–	DLDs: 7 years, 2 months–9 years, 3 months TDs: 7 years, 6 months–8 years, 11 months	8 years, 1 month	31	ns
24	30	67	–	DLDs: 24–32 months; TDs: 25–30 months	27.0 months	47	ns
25	14	31	(20, ASDs)	DLDs and TDs (experiment 1): 24.5–34.7 months; ASDs (experiment 2): 26.5–64.5 months	ns	30	ns
26	20	20	–	Overall: 4–8 years	6.5 years	30	ns
27	20	20	–	Overall: 4–5 years	57.70 months	45	3 (overall)
28	20	20	–	DLDs: 5 years, 1 month–6 years, 6 months; TDs: 5 years, 1 month–6 years, 7 months	5;9 years	30	ns
29	Time point 1: 20; time point 2: 15	Time point 1: 20; time point 2: 12	–	Overall: 5 years, 4 months–6 years, 6 months (time point 1); 6 years, 5 months–7 years, 7 months (time point 2)	5;10 years	30	ns
30	34	45	–	Overall: 5–10 years	Younger Group: 6.18 years; Older Group: 8.88 years	47	ns
31	20	20	–	Overall: 5 years, 7 months–10 years, 10 months	95.8 months	20	ns
32	30	26	–	ns	8.4 years	37	ns
33	34	45	–	Overall: younger group: 5 years, 0 month–7 years, 11 months; Older Group: 8 years, 0 months–10 years, 11 months	Younger Group: 6.18 years; Older Group: 8.88 years	47	ns

**FIGURE 2:**
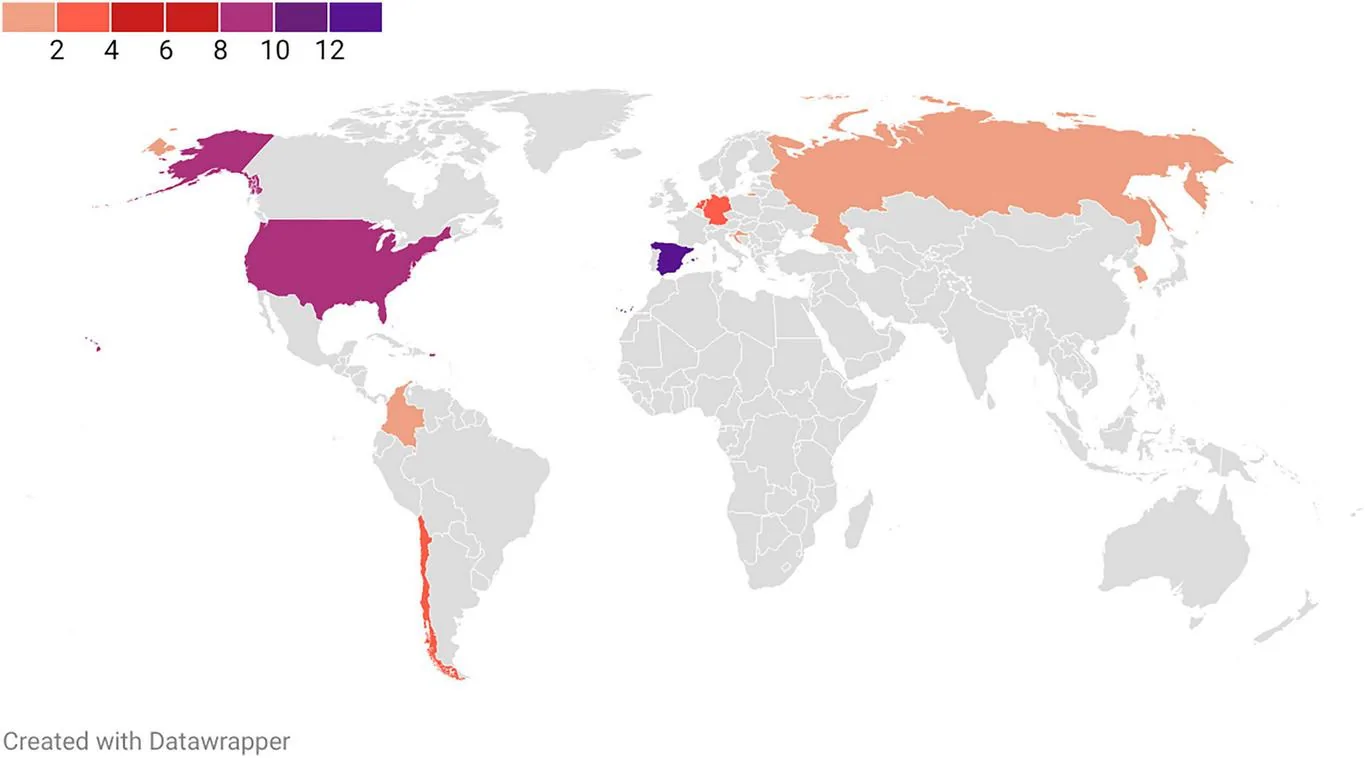
Visualization of geographical distribution. The color scale represents the number of studies (N).

The visualization of the geographic distribution was created with Datawrapper and is displayed in Figure 2.

### 3.2 Participant characteristics and sampling adequacy

The participants in the included studies were divided into DLD groups, control groups, and other groups. The control groups primarily included age-matched controls. Six studies also included linguistic control groups based on MLU-w (Mean Length of Utterance in Words). In addition, six studies incorporated adult control participants that served as language-expert control groups. Two studies featured ASD participants. Only one study McMurray et al. (2010) included non-specific-language-impaired groups and specific cognition-impaired groups.

The age range (0–18 years) of the participants with DLD aligned with the eligibility criteria. The mean age of such participants per experiment ranged from 18 months to 17 years. The mean age of age-matched controls (e.g., TD/ASD) corresponded to such a range, which strengthened the validity of the research designs among these reviewed articles.

The sample size of participants ranged from 14 to 106 per experiment. The adequacy of sampling was appraised based on three dimensions: the sampling strategy, the sample size justification, and the representativeness of the sample. The adequacy of the sampling strategy was mainly demonstrated in studies’ methods of recruiting samples and the samples’ relevance to the research question. The sample size justification corresponded with the quantitative feature of the samples, namely the feasibility of conducting statistical analysis. The representativeness of the sample concerned samples’ capability of representing the entirety of a particular group based on elements such as age and linguistic backgrounds.

### 3.3 Paradigms and experimental designs

All studies adopted experimental or quasi-experimental study designs and also incorporated between-subject designs except that by Coloma et al. (2024), which incorporated longitudinal features by examining participants from two time points.

Twenty-two out of 33 studies adopted the Visual World Paradigm (VWP). The participants’ eye movements are recorded for further analyses (Huettig et al., 2011). Such a paradigm solely relies on the participants’ tendency to look at the experimental displays while listening to corresponding audio stimuli. In this sense, participants, especially young children, do not have to perform meta-linguistic judgments that may be demanding and speech-process-affecting (Huettig et al., 2011). Hence, the VWP is suitable for examining the implicit responses of children with DLD in eye-tracking experiments. Other paradigms primarily include the look-while-listening paradigm (LWL), the cross-situational word-learning paradigm, quick incidental learning (QUIL), and the subphonemic mismatch paradigm.

### 3.4 Eye-tracking metrics and analytical practices

The technical specifications for the present systematic review are summarized in Table 5.

**TABLE 5:** Technical specifications of the present systematic review.

No.	Device/model	Sampling rate (Hz)	Calibration method	Screen size/resolution	Areas of interest (AOIs) (Y/N)	Time window/analysis windows (Y/N)
1	EyeLink II head-mounted eye tracking system	250	9-point	ns	N	N
2	Iriscom quick glance 2SH device	25	ns	15”, 1,280 × 800	Y	Y
3	SMI iView HiSpeed	500	13-point	ns	N	Y
4	Tobii T120	120	9-point	17”, 1,024 × 768	Y	Y
5	Iriscom QuickGlance 2SH	25	ns	15”, 1,280 × 800	Y	Y
6	Tobii T120	120	9-point	17”, 1,024 × 480	Y	Y
7	EyeLink 2000 remote	500	5-point	17”, ns	Y	Y
8	Tobii T120	120	9-point	17”, 1,024 × 768	Y	Y
9	EyeLink 2000 remote	500	ns	17”, ns	N	Y
10	Tobii 1750/Tobii T60	50/60	ns	17” (for both), ns	N	Y
11	SR research EyeLink 1000	500	9-point	17”, 1,280 × 1,024	Y	Y
12	Tobii T120	120	ns	17”, 1,024 × 768	N	Y
13	Infrared remote eye-tracking system (RED)	60	5-point	24”, 1,920 × 1,080	Y	N
14	SR research EyeLink 1,000 system	500	ns	15”, 1,280 × 720	Y	N
15	Tobii TX300	300	9-point	23”, 1,366 × 768	Y	Y
16	Tobii T120	120	ns	17”, 1,024 × 768	Y	Y
17	Tobii T120	120	ns	17”, ns	Y	Y
18	Tobii T120	120	9-point	17”, 1,024 × 768	Y	Y
19	EyeLink 1000 Plus	500	5-point	24”, 1,024 × 768	Y	Y
20	Tobii T120	120	ns	17”, 1,024 × 768	Y	Y
21	Tobii X3-120	120	ns	24”, 1,920 × 1,080	Y	Y
22	Tobii X3–120	120	ns	24”, ns	Y	Y
23	Tobii Pro X2-120 mobile	120	9-point	17”, ns	Y	Y
24	Tobii Pro X3-120 corneal reflection	120	ns	34.5 × 19.5 cm, 1,920 × 1,080	N	Y
25	Tobii T60 XL corneal refection eye-tracking monitor	ns	5-point	24”, ns	N	Y
26	Tobii TX300	300	9-point	23”, 1,920 × 1,080	Y	Y
27	EyeLink 1000 plus	500	5-point	24”, ns	Y	Y
28	EyeLink 1000 plus eye tracking system	500	5-point	24”, ns	Y	Y
29	The remote mode of the EyeLink 1000 Plus eye tracker system	500	5-point	24”, 1,024 × 768	Y	Y
30	Tobii Pro Nano 60 Hz	60	ns	21.5”, 1,920 × 1,080	Y	Y
31	EyeLink 1000 portable duo system	500	5-point	Either 17” (laptop) or 24” (monitor), both 1,920 × 1,080	Y	Y
32	EyeLink 1000 desktop-mounted video-based eye-tracking system	ns	9-point	22”, 1,680 × 1,050	Y	Y
33	Tobii Pro Nano 60 Hz	60	ns	21.5”, 1,920 × 1,080	Y	Y

Mak et al. (2017) conducted the experiments in both Russia and Netherlands and used divergent eye-trackers.

The studies primarily utilized images as visual stimuli. These studies incorporated Areas of Interest (AOIs) to identify participants’ fixation on these visual stimuli, such as target images and distractors. The AOIs in such studies corresponded to the images regarding location and size. Time windows were adopted by the majority of the studies for further analysis.

Notably, fixation proportions and the proportion of looks were used in 13 and 12 studies as the primary eye-tracking metric. Such a phenomenon was presumably due to the peculiarities of the DLD experiments in which the stimuli were limited to visuals instead of sentences. Proportion of looks and fixation proportions measured participants’ fixation on AOIs relative to other AOIs or non-AOI regions. Such metrics indicated participants’ visual attention to the corresponding stimulus. Other recorded metrics include fixation duration, reaction time, and latency time.

The reviewed studies employed statistical models to convert recorded data into interpretable findings. Specifically, seven studies utilized generalized linear mixed models (GLMM), while 13 studies incorporated linear mixed models (LMM). In addition, eight studies adopted analysis of variance (ANOVA). A subset of studies specified their distinctive models. For instance, that by Kueser et al. (2024) featured Bayesian mixed-effects models, that by Curtis et al. (2023a) implemented binomial logistic mixed-effects models and growth curve models, and that by Andreu et al. (2013b) applied multilevel logistic models with crossed-random effects.

### 3.5 Primary cognitive-linguistic outcomes assessed

The primary linguistic domains assessed in each study were recorded based on their respective research questions, aims, and findings. The recorded data were organized by construct using the data extraction sheet and were displayed in Tables 6, 7.

**TABLE 6:** Primarily assessed linguistic domains of the present systematic review.

No.	Lexical	Phonological	Morphological	Syntactic	Semantic	Pragmatic	Grammatical	Discourse
1	✓	–	–	–	–	–	–	–
2	–	–	–	✓	✓	–	–	✓
3	–	–	–	✓	✓	–	–	–
4	✓	–	–	–	–	–	–	–
5	✓	–	–	✓	✓	–	–	✓
6	–	✓	–	–	–	–	–	–
7	✓	–	–	–	✓	–	–	–
8	✓	–	–	–	✓	–	–	–
9	✓	✓	–	–	–	–	–	–
10	–	–	–	–	–	✓	–	✓
11	✓	✓	–	–	–	–	–	–
12	–	–	✓	–	–	–	–	–
13	✓	–	–	–	–	–	–	–
14	–	–	–	✓	✓	✓	✓	–
15	✓	–	–	–	✓	–	–	–
16	–	–	–	✓	✓	–	✓	–
17	✓	–	–	–	✓	–	–	–
18	–	–	✓	✓	–	–	✓	–
19	✓	✓	–	–	✓	–	–	–
20	–	–	✓	✓	–	–	✓	–
21	✓	✓	–	–	–	–	–	–
22	✓	–	–	–	✓	–	–	–
23	✓	–	–	–	✓	–	–	–
24	✓	–	–	–	✓	–	–	–
25	✓	–	–	–	✓	–	–	–
26	✓	✓	–	–	–	–	–	–
27	✓	–	✓	✓	✓	–	–	–
28	✓	–	–	–	✓	–	–	–
29	–	–	✓	✓	–	–	–	–
30	–	✓	–	–	–	✓	–	–
31	–	–	–	✓	✓	–	✓	–
32	✓	–	✓	✓	✓	–	✓	–
33	–	✓	–	✓	–	–	–	–

**TABLE 7:** Main findings of the present systematic review.

No.	Main findings
1	Adolescents with weaker language skills showed fewer looks to the target item and more fixations to the cohort and rhyme competitors, with behavioral data suggesting that variability in overall language ability may be associated with highly specific changes in word recognition.
2	During narrative production, children with SLI looked less frequently at the most semantically relevant areas of scenes than age-matched controls, albeit no differences were found in narrative comprehension. Furthermore, analyses of speech production further indicated that children with SLI retained less information during retelling and produced more semantic and grammatical errors than control children.
3	Children with SLI exhibit overall slower performance, while also suggesting that not all aspects of language are equally affected; specifically, children with SLI appear to be weaker in argument structure building, although the group showed slightly stronger anticipatory looks in the “semantic” condition.
4	All children exhibited an animacy effect in visual exploration before word completion, and that group differences between nouns and verbs emerged after the completion. Overall, nouns were recognized faster than verbs, and one-verb arguments were recognized faster than two- and three-verb arguments, whereas children with SLI were slower than their controls and especially in the recognition of three-argument verbs.
5	As the verb argument complexity increased, children with SLI made more omissions of obligatory arguments, particularly themes. Furthermore, compared to age-matched controls, this group looked less at the event zone during the first two seconds, and spent significantly less time looking at the theme zones. These differences between the groups were significant for three-argument verbs.
6	Children with SLI were impaired not only in their speech perception abilities, but also in their processing of the temporal coherence of auditory and visual speech information, showing poorer ability than typically developing peers to distinguish between temporally coherent and incoherent auditory and visual attributes of speech.
7	Adolescents with SLI displayed more diffuse patterns of fixation to the scene images, spent less time gazing at the image centers, and were more likely to fixate toward the image edges than their TD peers. Additionally, individuals with SLI differed in their fixation patterns to non-target items and in the spatial distribution of their fixation around the visual scene, suggesting that they may consider fewer lexical candidates as the sentence unfolds and instead restrict their fixations to highly likely sentence outcomes.
8	Despite their linguistic deficits, children with SLI performed relatively well on a real-time spoken language comprehension task requiring the mapping of perceived speech onto visual referents; nevertheless, their performance remained somewhat poorer than their age-matched peers.
9	Late-talking and typically developing 18-month-olds showed emerging group differences in the learning and interpretation of novel words, although both groups demonstrated comparable overall learning of novel word forms.
10	At least in the domain of discourse connectives, bilingual children with TLD (Typical Language Development) and monolingual children with SLI possess distinct profiles despite showing similar production profiles.
11	Typically developing listeners showed evidence of inhibition, reflected in greater interference for stimuli that briefly activated a competing word, whereas listeners with DLD did not exhibit this pattern; these findings suggest that language deficits in DLD may stem from a failure to engage lexical inhibition, potentially producing ripple effects across the language system.
12	Given sufficient processing time, both children with normal language acquisition and children with DLD were able to comprehend morphological markers within articles in simple sentence structures.
13	Typically developing children inferred word-referent mappings and strengthened these associations over time, whereas children with SLI had difficulty associating novel labels with novel objects, as reflected by reduced looking time to areas of interest. These findings provide insights into quick incidental learning of words in naturalistic contexts among children with and without SLI.
14	The study reported no differences between the clinical and control groups in how they resolved the tasks. In the first experiment, the age-matched control group showed shorter reaction time than the ASD group without language disorder, possibly because pronoun processing imposed a greater cognitive load on the latter group.
15	Both groups exhibited word recognition, although children with DLD shifted their gaze more slowly from distractors to targets and maintained longer looks to the target; within the DLD group, recognition was associated with expressive language scores, and children with DLD also showed a reduced prediction effect and slower shifts from verb onset.
16	Despite certain differences, both children with DLD and the control groups can comprehend prepositions and prepositional locutions in simple sentences in Spanish.
17	Both groups of children performed above chance level (25%) on the 4-AFC test following the CSSL task. However, children with DLD achieved significantly lower accuracy than typically developing children (37% vs. 48%), suggesting that they were able to learn words in non-sequential ambiguous statistical learning contexts, but less successfully than their TD peers.
18	Children with DLD showed a preserved capacity to comprehend Spanish verbal number morphology under the experimental conditions examined, as they were able to clearly distinguish correct from incorrect stimuli by the end of both tasks.
19	When a preview was provided, both groups were similarly attracted to cohort and semantic competitors and showed a preference for shape competitors over unrelated objects; however, this shape preference disappeared in the DLD group when no preview was available and when the shape competitor was presented alongside a semantic competitor, suggesting that children with DLD retrieve shape representations less effectively during word recognition than typically developing children.
20	Both children with DLD and children in the control groups were able to comprehend past future distinctions in simple sentence structures.
21	Both toddlers with typical expressive vocabularies and late talkers exhibited sensitivity to mispronunciations, suggesting that stored phonological representations of familiar words in both groups contain sufficient detail to detect minor pronunciation changes, at least in word initial position.
22	Despite having smaller expressive vocabularies, late talkers had encoded semantic relationships in their receptive vocabularies and were able to activate these relationships during real-time language comprehension.
23	Children with DLD were less proficient than typically developing children in learning word meanings from cross-situational statistics in an implicit task, although they were able to acquire word-referent mappings, they experienced greater difficulty and may require more input to reach similar levels of performance.
24	Late talkers demonstrated slower lexical processing, particularly of verbs; however, their successful use of familiar verbs to learn novel nouns suggests that this slower processing did not, at the group level, impair word learning in the task.
25	In experiment 1 of the study, late talkers and typically developing children did not differ in the proportion of verbs known, although late talkers required additional time to demonstrate this knowledge, with latency predicted by age rather than language abilities. In experiment 2, both accuracy and latency among autistic children were predicted by receptive language abilities.
26	Children with DLD demonstrated deficits in both phonological awareness and visual attention skills during linguistic and phonological processing tasks, and also exhibited reduced sensitivity in identifying phonological relationships such as rhyme.
27	Children with DLD can integrate event-probability information with local morphosyntax but failed to do so with distant morphosyntax. Furthermore, verb vocabulary size positively predicted the use of event-probability cues in both groups.
28	Lexical access to frequent words was as developed in monolingual preschoolers with DLD as in their TD peers, suggesting that semantic difficulties in this population are less severe in comprehension than production.
29	Children with DLD require more time than their age-matched peers to acquire and comprehend the morphological information of articles.
30	Compared with TD children, children with DLD showed a less clear preference to look at the target image following the presentation of bodily and prosodic cues. However, multimodal cues reduced their bias toward the literal interpretation of indirect requests.
31	The study suggests that mild visuospatial processing problems may co-occur with DLD, and that these difficulties are partly influenced by age and nonverbal cognitive ability.
32	Both children with DLD and TD children utilized predictive processing strategies based on gender agreement and semantic information when decoding noun phrases. However, children with DLD were able to use gender cues only when noun phrases were presented at a slower speech rate, and their predictive certainty remained below age-typical levels even under this condition.
33	Prosodic cues influenced children’s comprehension of phrasal structures across groups, whereas gestures provided no benefit beyond prosody. However, children with DLD showed difficulties integrating visual information, suggesting that they can rely on prosodic cues to support sentence comprehension while highlighting the importance of integrating multimodal cues in linguistic interactions.

## 4 Discussion

The present systematic review synthesizes evidence from 33 studies utilizing eye-tracking technology to investigate linguistic features in children with developmental language disorder (DLD). The review provides a comprehensive overview of the field by focusing on diverse linguistic domains, experimental designs, and linguistic mechanisms suggested by the findings of the included studies. Accordingly, the detailed aspects of such studies were extracted and analyzed. Such aspects include paradigms, metrics, analytical methods, and the primary linguistic outcomes assessed. In addition, to provide insights into future research directions, indexes such as the geographical distribution of studies were organized and further studied.

### 4.1 Geographic distribution and language of the studies

The findings of this review suggest that the majority of the research is concentrated in Europe and the United States, with a primary focus on English and Spanish as the languages for investigation. Two potential explanations may account for the prevalent use of Spanish in this field. First, Spanish is a global language that possesses a vast number of speakers and diverse dialects, prompting researchers to recruit adequate samples of participants with DLD. Second, Spanish is a language with distinctive features across various linguistic domains, making it particularly suitable for revealing differences between participants with DLD and their counterparts, such as TD children. For instance, Spanish has rich morphological features, including tense markers (e.g., future and past tenses) as well as gender and number agreement. Christou et al. (2022a) examined verbal tense morphology in children with DLD and emphasized that inflectional morphology may represent one of the most significant challenges for such children.

Future research could be conducted in countries such as Canada, Australia, and European nations beyond Spain to broaden the geographic and linguistic scope of the field. For instance, researchers may recruit bilingual and even multilingual participants with DLD from immigrant countries like Australia. In addition, researchers could also incorporate other non-alphabetic languages such as Japanese and Chinese to uncover DLD’s characteristics.

### 4.2 Methodological insights from included studies

The majority of the reviewed studies featured typically developing children and adults as the comparison groups, with only Horvath and Arunachalam (2023), and Moreno-Pérez et al. (2020) incorporating children with autism spectrum disorder (ASD). Such disorders are not segregated within the linguistic domain and often exhibit overlaps in speech, language, and communication (Bishop et al., 2017). Clinically, children with DLD and those with ASD often display similar language difficulties. For instance, researchers may observe common pragmatic difficulties in children with ASD or other neurodevelopmental disorders (Bishop et al., 2017). In addition, there may be notable differences between children with DLD and ASD in terms of language difficulties that require further investigation. On these accounts, future research would benefit from examining similarities and differences in linguistic deficits among children with DLD and ASD.

The included studies primarily employed the Visual World Paradigm (VWP). Among these studies, several modified the paradigm to achieve optimal investigations aligned with their specific research objectives. For instance, Giberga et al. (2025a,b) adopted a modified design that requires children to visually process images and a speaker positioned at the center of the screen. Such an approach enabled these studies to more effectively examine how children process multimodal cues. In addition to the VWP, several studies have used alternative paradigms suited to their research aims. For example, Broedelet et al. (2023) employed a cross-situational paradigm that investigated potential difficulties in children with DLD when learning word-referent pairs. Future research could incorporate a wider range of paradigms to investigate the linguistic abilities of children with DLD.

The sampling rate of eye-tracking devices represents the frequency at which the device records eye movements per second. A higher sampling rate provides greater temporal resolution, although overall data quality also depends on calibration accuracy, trackloss, participant compliance, and the suitability of the experimental setup. The sampling rate in the included studies ranged from 25 to 500 Hz, with the most common rate being 120 Hz. This variability indicates differences in temporal resolution across studies. Future research should select a sampling rate appropriate to the research questions, target measures, participant age, and available resources, while also reporting calibration accuracy and trackloss transparently.

Trackloss refers to the proportion of gaze samples that are not successfully recorded or retained for analysis. In child eye-tracking research, trackloss may arise from blinks, head movements, looking away from the stimulus, calibration instability, or hardware-related recording issues. Excessive trackloss can reduce the number of analyzable trials and affect the reliability of gaze-based measures. Therefore, future studies are encouraged to report trackloss rates and trial-exclusion criteria more transparently. Child-friendly experimental designs, shorter testing sessions, engaging calibration procedures, and appropriate eye-tracking setups may help improve gaze stability and reduce data loss.

Statistical models such as ANOVA, LMM, and GLMM were applied in the included studies for eye-tracking data analysis. A few studies also employed distinctive models that are tailored to their analytical approach. Future studies may consider utilizing complex statistical models according to their respective eye movement data.

### 4.3 Interpretation of main findings

The synthesis of the included studies reveals several recurring patterns in how children with developmental language disorder process linguistic information in real time. Although the experimental paradigms and linguistic domains varied across studies, the findings coherently suggest systematic differences between children with DLD and typically developing comparison groups regarding processing efficiency, predictive mechanisms, and cue reliance during language comprehension and production.

#### 4.3.1 Slower real-time processing in DLD

One of the most consistent findings across the reviewed studies is that children with DLD often demonstrate slower or less efficient real-time processing compared to typically developing counterparts. This pattern has been observed across multiple linguistic domains, including lexical access, morphosyntactic processing, and sentence interpretation. Eye-tracking measures frequently revealed delayed gaze shifts toward target stimuli or longer latencies in processing linguistic information.

Importantly, several studies indicated that children with DLD were ultimately able to reach correct interpretations but required longer processing time. Such findings suggest that the primary difficulty may lie in processing efficiency rather than the complete absence of linguistic knowledge. In other words, children with DLD may possess relevant linguistic representations but experience difficulties accessing or integrating them rapidly during real-time processing.

#### 4.3.2 Reduced predictive processing

Another prominent pattern concerns reduced or delayed predictive processing in children with DLD. In various eye-tracking studies, typically developing children exhibited anticipatory eye movements based on linguistic cues such as verb semantics or morphosyntactic markers. In contrast, children with DLD often showed weaker or delayed prediction effects.

Predictive processing is crucial in efficient language comprehension, as this ability allows listeners to anticipate upcoming linguistic information and reduce processing load. The reduced predictive ability observed in children with DLD may therefore contribute to their slower processing patterns during language comprehension tasks. These findings are consistent with studies suggesting that children with DLD may experience difficulties integrating multiple sources of linguistic information rapidly during online processing.

#### 4.3.3 Reliance on semantic cues

A further pattern emerging from the reviewed studies is that children with DLD may rely more heavily on semantic cues rather than morphosyntactic information during language processing. This pattern is particularly evident in Studies 3 and 27. Study 3 reported stronger anticipatory looks in the semantic condition but weaker argument-structure building. Study 27 showed that children with DLD could integrate event-probability information with local morphosyntax but struggled with distant morphosyntax.

This pattern suggests that children with DLD may rely more heavily on meaning-based information when morphosyntactic cue processing is demanding. Such reliance on semantic cues may allow children with DLD to achieve relatively accurate comprehension outcomes in some tasks despite difficulties in processing grammatical morphology or syntactic structures in real time.

#### 4.3.4 Cross-domain comparison

Across linguistic domains, eye-tracking evidence suggests that DLD is characterized by a pattern of increasing difficulty as processing demands increase. This suggests that children with DLD do not show the same degree of language processing difficulty across all linguistic domains.

In the lexical-semantic domain, studies suggest that children with DLD are often able to recognize familiar words and access lexical-semantic information but do so less efficiently than their TD peers. For instance, Study 24 reported slower lexical processing in the DLD group, particularly for verbs, although this processing inefficiency did not impede word learning in the experiment. Similarly, Experiment 1 of Study 25 showed that children with DLD may require additional time to demonstrate verb knowledge. In addition, Study 22 suggests that late talkers were able to encode semantic relationships in receptive vocabulary. These findings indicate that children with DLD may be less efficient regarding the real-time access and use of lexical-semantic knowledge.

Findings in the phonological domain are more mixed. Study 21 suggests that late talkers possess detailed phonological representations and are sensitive to mispronunciations. However, Studies 6 and 26 reported that this group may have difficulties in auditory-visual speech synchrony, phonological awareness, and sensitivity to phonological relationships such as rhymes. These findings suggest that phonological difficulties in children with DLD may reflect reduced efficiency in using phonological information during real-time processing, particularly when tasks require the integration of auditory and visual speech cues or the detection of subtle phonological differences.

Findings in the morphological and grammatical domains suggest that children with DLD may retain morphosyntactic knowledge but experience difficulties using such information efficiently in real time. Studies 12, 16, 18, and 20 showed that children with DLD could comprehend Spanish articles, prepositions and prepositional locutions, verbal morphology, and past-future differences in simple sentences. However, Study 29 suggests that they required additional time to acquire and comprehend morphological information in articles. Furthermore, Studies 32 and 15 reported that the DLD group may be less efficient in predictive processing. These findings showed a pattern similar to that observed in the lexical-semantic domain, suggesting reduced processing efficiency.

Studies regarding syntax, argument structure, and discourse indicate that children with DLD may encounter greater integration demands. Study 2 showed that children with DLD retained less information, produced more semantic and syntactic errors during narrative retelling, and looked less often at semantically relevant regions. Studies 3–5 further suggest weaker argument-structure processing, including weaker argument-structure building, slower verb recognition, more omissions of obligatory arguments, and reduced looks to event and theme zones. Consistent with this pattern, Study 33 reported that children with DLD encountered difficulties integrating visual information during sentence comprehension. In contrast, Studies 12, 16, 18, and 20 suggest relatively adequate comprehension in simple sentence-processing tasks. These findings indicate that children with DLD may encounter processing difficulties as complexity and integration load increase.

Studies on pragmatic and multimodal cues further extended this integration pattern. Evidence from Studies 30 and 33 suggests that children with DLD were able to use prosodic cues to support comprehension but showed less clear use of multimodal information, including weaker target-image preferences following bodily and prosodic cues and difficulty integrating visual information during sentence comprehension. These findings suggest that children with DLD can utilize supportive cues such as prosody, but their difficulties become more apparent when comprehension requires the coordination of multiple linguistic, pragmatic, and visual cues.

Taken together, these findings suggest that difficulties experienced by children with DLD are most consistently observed in the efficiency of processing. Across lexical, morphological, syntactic, and pragmatic domains, these children tend to process information less efficiently than their TD peers, and these processing difficulties become apparent as linguistic demands increase, especially when tasks involve morphosyntactic prediction, argument-structure complexity, discourse integration, or multimodal cue coordination. Lastly, semantic cues appear to be more accessible than morphosyntactic and multimodal cues. Therefore, eye-tracking technology may provide useful evidence for identifying cross-domain processing limitations in children with DLD.

#### 4.3.5 Theoretical interpretation

The recurring patterns identified in the present systematic review can be interpreted in relation to current theoretical accounts of DLD.

The slower real-time processing observed in the reviewed studies is consistent with Leonard’s (2014) processing-limitation account of SLI/DLD. Leonard’s synthesis treats many linguistic and nonlinguistic findings in SLI as evidence for limitations in information-processing capacity, which can be conceptualized regarding space, energy, and time. Time restriction is particularly relevant to real-time language comprehension, because when linguistic information is not processed rapidly enough, it may become vulnerable to decay or interference from subsequent input. Within this framework, the generalized-slowing hypothesis proposes that children with SLI respond more slowly than age-matched TD peers across processing tasks, with more apparent delays when tasks require additional cognitive operations. Leonard reviewed evidence of slower reaction times in tasks such as sentence completion and grammaticality judgments.

Therefore, this account provides a framework for interpreting delayed gaze shifts and longer processing latencies in children with DLD. Furthermore, these delays suggest that children with DLD may experience difficulties in accessing, maintaining, and integrating linguistic representations in real-time processing contexts. However, Leonard emphasized that although many children with SLI/DLD exhibit reduced speed of processing across linguistic and nonlinguistic areas, this reduction is not evident in every child with SLI/DLD and may vary across domains and within domains.

The reduced or delayed predictive effects observed in children with DLD can be interpreted within Pickering and Garrod’s (2013) integrated theory of language production and comprehension, which proposes that comprehension and production are closely interwoven. According to this account, speakers and listeners may generate predictions at multiple levels of linguistic representation, such as semantics, syntax, and phonology. Within this framework, their forward modeling account suggests that predicted perceptual representations can become available before actual perceptual feedback. Discrepancies can then be used to adjust subsequent processing, because relying exclusively on reafferent feedback would be too slow for online corrective actions.

This framework provides useful theoretical insights into weaker or delayed anticipatory eye movements in children with DLD. Such patterns may suggest that these children are less efficient at using linguistic cues actively during online comprehension.

The reliance on semantic cues observed in children with DLD can also be interpreted within the Competition Model proposed by Bates and MacWhinney (1989). This model suggests that sentence comprehension involves competition among multiple cues, including information such as word order, animacy, grammatical morphology, and prosodic contrasts. Cue use is shaped by the relative value of these cues, with listeners tending to rely more on cues that are relatively available, reliable, and readily accessible during real-time processing. Furthermore, Bates and MacWhinney (1989) suggest that children may form tentative interpretations during sentence comprehension by relying on word order and semantic information, rather than holding sentence elements in memory to determine whether agreement cues are available.

From this perspective, children with DLD may rely more heavily on semantic cues because these cues are often more immediately accessible than morphosyntactic cues.

An alternative account, which concerns the developmental status of grammatical representations, is provided by Rice and Wexler’s (1996) Extended Optional Infinitive (EOI) model. It proposes that “the broader grammatical scaffolding in children with SLI is largely intact, whereas selected areas of grammatical specification remain vulnerable” (p.1254). Rather than locating the primary difficulty exclusively in language processing, the EOI model posits that the obligatory status of tense and finiteness marking may remain underdeveloped or underspecified for an unusually prolonged developmental period. Consequently, children with DLD may treat overt finiteness markers as optional in contexts in which adult grammar requires them. Longitudinal evidence further indicates that these children may continue to display optional finiteness marking through 15 years of age (Rice et al., 2009).

From this perspective, delayed or reduced eye-movement sensitivity to morphosyntactic cues may reflect not solely inefficient access to otherwise intact linguistic information but also delayed or vulnerable grammatical representations. Nevertheless, eye-tracking evidence alone cannot determine whether such patterns arise from processing inefficiency, representational weakness, or a dynamic interaction between the two.

#### 4.3.6 Developmental trajectories and persistence

The developmental persistence of these processing and linguistic differences cannot be determined conclusively from the current eye-tracking evidence, because most included studies were cross-sectional. One exception was Study 29, which followed the same participants longitudinally and reassessed the children approximately 1 year after the initial experiment. It found that the processing of participants with DLD remained slower and weaker than that of their age-matched typically developing peers, which suggests a persisting delay relative to typical development. Although this study provides some evidence concerning developmental change over time, a single longitudinal study is insufficient to establish whether the processing differences observed in children with DLD eventually diminish, persist, or change qualitatively with age. Complementary longitudinal evidence further suggests that selected grammatical difficulties in children with DLD may remain detectable into adolescence (Rice et al., 2009). Future longitudinal eye-tracking research is therefore needed to distinguish between developmental delays that gradually diminish and more persistent characteristics of DLD.

## 5 Conclusion

### 5.1 Overall contribution

The present systematic review aimed to offer a comprehensive understanding of studies using eye-tracking technology to examine language processing in children with developmental language disorder. Specifically, the review addressed the predefined questions by extracting, analyzing, and synthesizing data regarding participant sampling, methodological designs, and the primary linguistic outcomes assessed in the included studies. Additionally, the review focused on such studies’ fundamental information such as geographical distribution.

The review suggests that eye-tracking devices could provide a sensitive, fine-grained lens into real-time language processing in children with DLD. Furthermore, the review identified broadly consistent patterns in the language processing of children with DLD. The review also provided suggestions for future studies to potentially facilitate research quality. Such suggestions ranged from sampling adequacy to the use of statistical models.

### 5.2 Implications

The reviewed evidence suggests that eye-tracking studies may provide useful insights into real-time language processing in children with DLD. Across the included studies, children with DLD appeared to show differences in processing efficiency, predictive integration, and competition resolution, although the strength and consistency of these patterns varied across linguistic domains. These findings are compatible with the possibility that some children may possess relevant linguistic representations but experience difficulty accessing, integrating, or updating such information efficiently during online processing. Furthermore, several studies suggest that children with DLD may rely more heavily on semantic or event-probability cues when morphosyntactic cue processing is demanding. Given the heterogeneity of the included studies, these interpretations should be treated as tentative and should be tested further in future research.

This systematic review is subject to several limitations. Firstly, the review exclusively considered peer-reviewed journal articles in alignment with its inclusion criteria. Such an approach may underestimate the potential of relevant literature such as conference papers or published books. Further studies may also refer to these resources to establish a comprehensive understanding. Additionally, the review focused on studies written in English. Journal articles written in other languages such as Korean were screened during the article selection process. Future researchers may approach such articles with assistance such as professional translation tools. Furthermore, the included studies were highly heterogeneous regarding participant characteristics, linguistic domains, experimental paradigms, eye-tracking metrics, and statistical reporting practices. This heterogeneity limited the feasibility of quantitative synthesis and requires caution when generalizing the findings across populations, languages, and linguistic domains. Lastly, the reporting quality of the included studies varied in this review. Details of these studies such as sampling adequacy, addressed confounders, and completeness of outcome data may not be consistently comprehensive. Researchers are suggested to approach this review critically. Despite the addressed limitations, this systematic review offers critical and precise insights into the application of eye-tracking tools to the examination of linguistic features in children with DLD.

### 5.3 Closing statement

At present, studies using eye-tracking technology to investigate linguistic features of children with DLD remain underrepresented and scattered. Further research is encouraged to employ eye-tracking devices when examining children with DLD. Furthermore, future studies could focus on multilingual participants, non-alphabetic languages, diverse paradigms and statistical analysis approaches, appropriate sampling rates of eye-tracking devices, and transparent data-quality reporting to enable cumulative theoretical advancement.

## Data Availability

The original contributions presented in this study are included in this article/supplementary material, further inquiries can be directed to the corresponding author.

## References

[B1] Ahufinger N. Guerra E. Ferinu L. Andreu L. Sanz-Torrent M. (2021). Cross-situational statistical learning in children with developmental language disorder. *Lang. Cogn. Neurosci.* 36 1180–1200. 10.1080/23273798.2021.1922723

[B2] Andreu L. Sanz-Torrent M. Guàrdia-Olmos J. (2012). Auditory word recognition of nouns and verbs in children with specific language impairment (SLI). *J. Commun. Disord*. 45 20–34. 10.1016/j.jcomdis.2011.09.003 22055614

[B3] Andreu L. Sanz-Torrent M. Olmos J. G. Macwhinney B. (2013a). The formulation of argument structure in SLI: An eye-movement study. *Clin. Linguist. Phon*. 27 111–133. 10.3109/02699206.2012.751623 23294226 PMC4073311

[B4] Andreu L. Sanz-Torrent M. Trueswell J. C. (2013b). Anticipatory sentence processing in children with specific language impairment: Evidence from eye movements during listening. *Applied Psycholinguistics* 34 5–44. 10.1017/S0142716411000592

[B5] Andreu L. Sanz-Torrent M. Guàrdia Olmos J. Macwhinney B. (2011). Narrative comprehension and production in children with SLI: An eye movement study. *Clin. Linguist. Phon*. 25 767–783. 10.3109/02699206.2011.565542 21453036 PMC4106358

[B6] Bahn D. Türk D. D. Tsenkova N. Schwarzer G. Võ M. L. Kauschke C. (2025). Processing of scene-grammar inconsistencies in children with developmental language disorder-insights from implicit and explicit measures. *Brain Sci*. 15:139. 10.3390/brainsci15020139 40002472 PMC11852763

[B7] Bates E. MacWhinney B. (1989). “Functionalism and the competition model,” in *The crosslinguistic study of sentence processing*, eds MacWhinney B. Bate E. (New York, NY: Cambridge University Press), 3–76.

[B8] Bishop D. V. M. Snowling M. J. Thompson P. A. Greenhalgh T. (2017). Phase 2 of CATALISE: a multinational and multidisciplinary delphi consensus study of problems with language development: Terminology. *J. Child. Psychol. Psychiatry*. 58 1068–1080. 10.1111/jcpp.12721 28369935 PMC5638113

[B9] Borovsky A. Burns E. Elman J. L. Evans J. L. (2013). Lexical activation during sentence comprehension in adolescents with history of specific language impairment. *J. Commun. Disord*. 46 413–427. 10.1016/j.jcomdis.2013.09.001 24099807 PMC4634526

[B10] Broedelet I. Boersma P. Rispens J. (2023). Implicit cross-situational word learning in children with and without developmental language disorder and its relation to lexical-semantic knowledge. *Front. Comm.* 8 10.3389/fcomm.2023.1021654

[B11] Cholewa J. Kirschenkern A. Steinke F. Günther T. (2025). Predictive use of grammatical gender during noun phrase decoding: An eye-tracking study with german children with developmental Language disorder. *J. Speech Lang. Hear Res*. 68 1056–1074. 10.1044/2024_JSLHR-24-00389 39913267

[B12] Christou S. Andreu L. Coloma C. J. Guerra E. Araya C. Rodriguez-Ferreiro J. et al. (2022a). Insights from real-time comprehension of spanish verbal tense in children with developmental language disorder:An eye-tracking study. *Applied Psycholinguistics* 43 641–662. 10.1017/S0142716422000042

[B13] Christou S. Coloma C. J. Andreu L. Guerra E. Araya C. Rodriguez-Ferreiro J. et al. (2022b). Online comprehension of verbal number morphology in children with developmental language disorder: An eye-tracking study. *J. Speech Lang Hear Res*. 65 4181–4204. 10.1044/2022_JSLHR-21-00591 36327553

[B14] Christou S. Guerra E. Coloma C. J. Andreu Barrachina L. Araya C. Rodriguez-Ferreiro J. et al. (2020). Real time comprehension of Spanish articles in children with developmental language disorder: Empirical evidence from eye movements. *J. Commun. Disord*. 87:106027. 10.1016/j.jcomdis.2020.106027 32652330

[B15] Christou S. Sanz-Torrent M. Coloma C. J. Guerra E. Araya C. Andreu L. (2021). Real-time comprehension of Spanish prepositions and prepositional locutions in bilingual children with developmental language disorder: A study based on eye-movement evidence. *Int. J. Lang. Commun. Disord*. 56 51–71. 10.1111/1460-6984.12581 33112042

[B16] Chung H. Yim D. (2020). Quick incidental learning of words by children with and without specific language impairment: An eye-tracking study. *Comm. Sci. Disord.* 25 499–516. 10.12963/csd.20715

[B17] Coloma C. J. Guerra E. De Barbieri Z. Helo A. (2024). Article comprehension in monolingual Spanish-speaking children with developmental language disorder: A longitudinal eye tracking study. *Int. J. Speech Lang. Pathol*. 26 105–117. 10.1080/17549507.2023.2167235 36647757

[B18] Conklin K. Pellicer-Sánchez A. (2016). Using eye-tracking in applied linguistics and second language research. *Second Lang. Res.* 32, 453–467. 10.1177/0267658316637401

[B19] Conti-Ramsden G. Durkin K. (2012). Postschool educational and employment experiences of young people with specific language impairment. *Lang. Speech Hear Serv. Sch*. 43 507–520. 10.1044/0161-1461(2012/11-0067) 22826369

[B20] Curtis P. R. Estabrook R. Roberts M. Y. Weisleder A. (2023a). Sensitivity to semantic relationships in U.S. monolingual english-speaking typical talkers and late talkers. *J. Speech Lang. Hear Res*. 66 2404–2420. 10.1044/2023_JSLHR-22-00563 37339002 PMC10468120

[B21] Curtis P. R. Estabrook R. Roberts M. Y. Weisleder A. (2023b). Specificity of phonological representations in U.S. *Infancy*. 28 771–792. 10.1111/infa.12536 36939533 PMC11908411

[B22] Ekström A. Sandgren O. Sahlén B. Samuelsson C. (2023). ‘It depends on who I’m with’: How young people with developmental language disorder describe their experiences of language and communication in school. *Int J Lang Commun. Disord*. 58 1168–1181. 10.1111/1460-6984.12850 36703539

[B23] Ellis E. M. Borovsky A. Elman J. L. Evans J. L. (2015). Novel word learning: An eye-tracking study. 58 143–157. 10.1016/j.jcomdis.2015.06.011 26188415 PMC4659719

[B24] Giberga A. Guerra E. Ahufinger N. Igualada A. Aguilera M. Esteve-Gibert N. (2025a). How children with and without developmental language disorder use prosody and gestures to process phrasal ambiguities. *Languages* 10:61. 10.3390/languages10040061 40138883

[B25] Giberga A. Guerra E. Ahufinger N. Igualada A. Aguilera M. Esteve-Gibert N. (2025b). Prosody and gestures help pragmatic processing in children with Developmental Language Disorder. *J. Commun. Disord*. 115:106525. 10.1016/j.jcomdis.2025.106525 40138883

[B26] Guerra E. Coloma C. J. Helo A. (2024). Lexical-semantic processing in preschoolers with Developmental Language Disorder: an eye tracking study. *Front. Psychol*. 15:1338517. 10.3389/fpsyg.2024.1338517 38807960 PMC11131166

[B27] Helo A. Guerra E. Coloma C. J. Reyes M. A. Rämä P. (2021). Objects shape activation during spoken word recognition in preschoolers with typical and atypical language development: Aneye-tracking study. *Lang. Learn. Develop* 18 324–351. 10.1080/15475441.2021.1977643

[B28] Hong Q. N. Fàbregues S. Bartlett G. Boardman F. Cargo M. Dagenais P. et al. (2018). The Mixed Methods Appraisal Tool (MMAT) version 2018 for information professionals and researchers. *Educ. Inf.* 34, 285–291. 10.3233/EFI-180221

[B29] Horvath S. Arunachalam S. (2023). Assessing receptive verb knowledge in late talkers and autistic children: advances and cautionary tales. *J. Neurodev. Disord*. 15:44. 10.1186/s11689-023-09512-x 38087233 PMC10717976

[B30] Hu X. Aryadoust V. (2024). A systematic review of eye-tracking technology in second language research. *Languages* 9:141. 10.3390/languages9040141

[B31] Huettig F. Rommers J. Meyer A. S. (2011). Using the visual world paradigm to study language processing: a review and critical evaluation. *Acta. Psychol.* 137 151–171. 10.1016/j.actpsy.2010.11.003 21288498

[B32] Kornilov S. A. Magnuson J. S. Rakhlin N. Landi N. Grigorenko E. L. (2015). Lexical processing deficits in children with developmental language disorder: An event-related potentials study. *Dev. Psychopathol*. 27 459–476. 10.1017/S0954579415000097 25997765 PMC4606961

[B33] Kueser J. B. Borovsky A. Deevy P. Muezzinoglu M. Outzen C. Leonard L. B. (2024). Verb vocabulary supports event probability use in developmental language disorder. *J. Speech Lang. Hear Res*. 67 1490–1513. 10.1044/2024_JSLHR-23-00600 38573844 PMC11087084

[B34] Lara-Díaz M. F. Costanza J. Aponte Rippe Y. (2024). Visual attention and phonological processing in children with developmental language disorder. *Front. Comm* 9. 10.3389/fcomm.2024.1386279

[B35] LaTourrette A. Waxman S. Wakschlag L. S. Norton E. S. Weisleder A. (2023). From recognizing known words to learning new ones: comparing online speech processing in typically developing and late-talking 2-year-olds. *J. Speech Lang. Hear Res*. 66 1658–1677. 10.1044/2023_JSLHR-22-00580 36989138 PMC10457094

[B36] Law J. Rush R. Schoon I. Parsons S. (2009). Modeling developmental language difficulties from school entry into adulthood: literacy, mental health, and employment outcomes. *J. Speech Lang. Hear Res*. 52 1401–1416. 10.1044/1092-4388(2009/08-0142) 19951922

[B37] Leonard L. B. (2014). *Children with Specific Language Impairment (2nd ed.).* Cambridge, MA: The MIT Press, 10.7551/mitpress/9152.001.0001

[B38] Leonard L. B. Eyer J. A. Bedore L. M. Grela B. G. (1997). Three accounts of the grammatical morpheme difficulties of English-speaking children with specific language impairment. *J. Speech Lang. Hear Res*. 40 741–753. 10.1044/jslhr.4004.741 9263940

[B39] Mak W. M. Tribushinina E. Lomako J. Gagarina N. Abrosova E. Sanders T. (2017). Connective processing by bilingual children and monolinguals with specific language impairment: distinct profiles. *J. Child Lang*. 44 329–345. 10.1017/S0305000915000860 26847820

[B40] McGregor K. K. (2020). How we fail children with developmental language disorder. *Lang Speech Hear Serv. Sch*. 51 981–992. 10.1044/2020_LSHSS-20-00003 32755505 PMC7842848

[B41] McMurray B. Klein-Packard J. Tomblin J. B. (2019). A real-time mechanism underlying lexical deficits in developmental language disorder: Between-word inhibition. *Cognition*. 191:104000. 10.1016/j.cognition.2019.06.012 31234114 PMC6988176

[B42] McMurray B. Samelson V. M. Lee S. H. Tomblin J. B. (2010). Individual differences in online spoken word recognition: Implications for SLI. *Cogn. Psychol.* 60 1–39. 10.1016/j.cogpsych.2009.06.003 19836014 PMC3523704

[B43] Moreno-Pérez F. J. Rodríguez-Ortiz I. R. Tavares G. Saldaña D. (2020). Comprehending reflexive and clitic constructions in children with autism spectrum disorder and developmental language disorder. *Int. J. Lang Commun. Disord*. 55 884–898. 10.1111/1460-6984.12568 32844517

[B44] Norbury C. F. Gooch D. Wray C. Baird G. Charman T. Simonoff E. et al. (2016). The impact of nonverbal ability on prevalence and clinical presentation of language disorder: evidence from a population study. *J. Child Psychol. Psychiatry*. 57 1247–1257. 10.1111/jcpp.12573 27184709 PMC5082564

[B45] Palmović M. Jušić I. (2011). Anticipatory gaze and argument structure building in children with specific language impairment. *Acta. Linguistica. Hungarica* 58 108–119. 10.1556/aling.58.2011.1-2.6 PMC3577096 23440891

[B46] Pickering M. J. Garrod S. (2013). An integrated theory of language production and comprehension. *Behav. Brain Sci*. 36 329–347. 10.1017/S0140525X12001495 23789620

[B47] Pons F. Andreu L. Sanz-Torrent M. Buil-Legaz L. Lewkowicz D. J. (2013). Perception of audio-visual speech synchrony in Spanish-speaking children with and without specific language impairment. *J. Child Lang*. 40 687–700. 10.1017/S0305000912000189 22874648 PMC3954717

[B48] Rice M. L. Wexler K. (1996). Toward tense as a clinical marker of specific language impairment in English-speaking children. *J. Speech Hear Res*. 39 1239–1257. 10.1044/jshr.3906.1239 8959609

[B49] Rice M. L. Hoffman L. Wexler K. (2009). Judgments of omitted BE and DO in questions as extended finiteness clinical markers of specific language impairment (SLI) to 15 years: a study of growth and asymptote. *J. Speech Lang. Hear Res*. 52 1417–1433. 10.1044/1092-4388(2009/08-0171) 19786705 PMC2787761

[B50] Royle P. Courteau E. (2014). “Language processing in children with specific language impairment: a review of event-related potential studies,” in *Language Processing: New Research*, eds Klein L. T. Amato V. (Hauppauge, NY: Nova Science Publishers), 33–36.

[B51] Speech and Language UK (n.d.). *Developmental language disorder (DLD) awareness.* London: Speech and Language UK: Changing Young Lives.

[B52] Toki E. I. (2024). Using eye-tracking to assess dyslexia: A systematic review of emerging evidence. *Edu. Sci.* 14 1256. 10.3390/educsci14111256

[B53] van Alphen P. Brouwer S. Davids N. Dijkstra E. Fikkert P. (2021). Word recognition and word prediction in preschoolers with (a suspicion of) a developmental language disorder: evidence from eye tracking. *J. Speech Lang. Hear Res*. 64 2005–2021. 10.1044/2021_JSLHR-20-00227 34019773

[B54] World Health Organization. (2025). *ICD-11 for mortality and morbidity statistics.* Geneva: World Health Organization.

